# Draft genome sequences of *Pseudomonas amygdali* pv. *loropetali* pathotype strain DSM 105780 ^PT^, isolated from Florida

**DOI:** 10.1099/acmi.0.000423

**Published:** 2022-09-08

**Authors:** Apekshya Parajuli, Carrie L. Harmon, Gerald V. Minsavage, Debra D. Jones, Sujan Timilsina, Mathews L. Paret, Jeffrey B. Jones

**Affiliations:** ^1^​ Plant Pathology Department, University of Florida, Gainesville, FL, USA; ^2^​ North Florida Research and Education Center, Plant Pathology Department, University of Florida, Quincy, FL, USA; ^3^​ Plant Diagnostic Center, Plant Pathology Department, University of Florida, Gainesville, FL, USA; ^4^​ Florida Department of Agriculture and Consumer Services, Division of Plant Industry, Gainesville, FL, USA

## Abstract

The pathogen that causes stem gall in *Loropetalum chinense* was first identified in Florida and Alabama in 2018 and named *Pseudomonas amygdali* pv. *loropetali*. We report the genome sequence of the pathotype strain of this pathogen, *Pseudomonas amygdali* pv. *loropetali* DSM105780 ^PT^.

## Announcement

*Pseudomonas amygdali* is a member of the *Pseudomonas syringae* complex and was initially identified as the cause of hyperplastic bacterial canker of almond (*Prunus communis* Arc) [1]. A novel pathovar, *Pseudomonas amygdali* pv. *loropetali* pv. nov, reported to cause stem galls on *Loropetalum chinense*, was isolated in Florida and Alabama [2]. *L. chinensis* is a hardwood species often used as firewood in mountainous part of southern China, whose individual flowers are clustered, covering the branches blooming like fireworks [3]. The strain of *Pseudomonas amygdali* pv. *loropetali* pv. nov, PDC13-208, isolated from Florida in 2012, was designated as the pathotype strain and deposited in DSM culture collection as DSM 105780^PT^. Harmon *et al*. (2018) identified this organism as a pathovar within *P. amygdali* based on multi-locus sequence analysis (MLSA) [2]. In order to corroborate the MLSA analysis on placement within this species and to understand its relatedness with other members of the *Pseudomonas syringae* complex, we applied next-generation sequencing to clarify the whole-genome sequence of the pathotype strain.

The bacterial strain was grown for 24 h at 28 °C in nutrient broth (BBL; Becton, Dickinson and Co., Franklin Lakes, NJ) and DNA was extracted with a Wizard genomic DNA purification kit (Promega, Chicago, IL). The DNA was sent to Microbial Genome Sequencing Center (Pittsburgh, PA) for library preparation and genome sequencing. A NextSeq 550 system (Illumina, San Diego, CA) was used to produce 151 bp end reads. The paired-end reads were assembled using SPAdes v. 3.15.3 and ‘--—careful’ switch [4]. Contigs smaller than 500 bp and with less than 2.0 of K-mer coverage were filtered using script filter-spades.py^1^. Trim Galore!^2^ v. 0.6.5 with default parameters was used to remove the adaptors from the paired-end reads and generate validated read files. Alignment of filtered contigs and remaining steps were performed as described by Fulton *et al*. (2020) [5].

Sequencing and genome statistics of the strain were calculated using Python and R scripts. R packages used were ape [6] and adegenet [7]. The draft genome comprises 314 contigs, with a G+C content of 58.068 %, N50 length of 73 733, sequencing genome coverage of 83.62×, and genome size of 6.35 Mb.

**Fig. 1.**
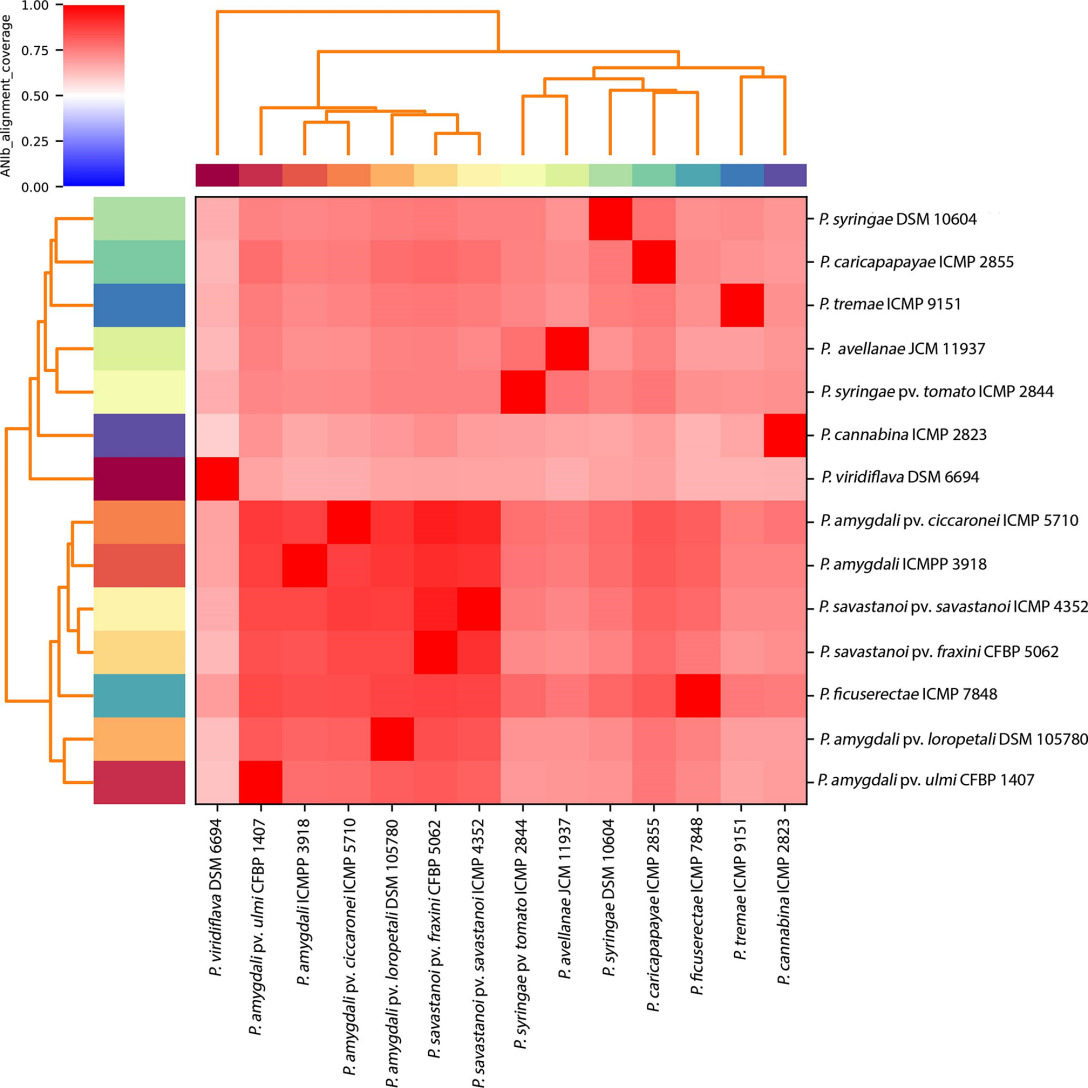
Average nucleotide identity (ANI) among members of *Pseudomonas syringae* complex. The ANI was calculated using the pyani program after blastn alignment. Only regions present in all genomes were used in the ANI calculation.

Average nucleotide identity (ANI) has been described as one of the best alternatives to DNA–DNA hybridization for prokaryotic species circumscriptions to the gold standard in which an ANI greater than 95–96 % can be used as a cut-off for considering a new prokaryotic species [8]. Pairwise ANIs of the strains and members of *P. syringae* complex were calculated (Table 1) and visualized (Fig. 1) using Python3 module Pyani 0.2.11^3^. Pairwise ANIs of the studied strain (* P. amygdali* pv. *loropetali* DSM 105780 ^PT^) were at least 98.1996 % within *P. amygdali* pathovars*,* including the *P. amygdali* type strain (ICMP 3198, ATCC 33050, DSM7298, NCPPB 3033) and 87.21 % with the type strain of *P. syringae* (DSM10604 ^T^) and the pathotype strain of *P. syringae* pv. *tomato* (ICMP 2844). Pairwise ANI of the strain DSM 105780^PT^ was at least 98.5 % with type strains of *P. savastanoi* pv. *fraxini* and *P. savastanoi* pv. *savastanoi,* and at least 97.4  % with the type strain of *P. ficuserectae* (ICMP7848).

**Table 1.** Comparison of percent average nucleotide identities of *Pseudomonas amygdali* pv. *loropetali* strain DSM 105780 ^PT^ and members of *P. amygadali* and *P. syringae* complex

	* P. viridiflava* DSM6694^T^	* P. ficuserectae * ICMP7848 ^T^	* P. caricapapayae* ICMP2855 ^T^	* P. amygdali* pv. *loropetali* DSM 105780 ^PT^	* P. tremae* ICMP9151 ^T^	* P. amygdali* ICMPP3918 ^T^	* P. amygdali* pv. *ciccaronei* ICMP5710 ^PT^	* P. avellanae* JCM11937 ^T^	* P. cannabina* ICMP2823 ^T^	* P. amygdali* pv. *ulmi* CFBP1407 ^PT^	* P. savastanoi* pv. *fraxini* CFBP 5062 ^PT^	* P. syringae* pv. *tomato* ICMP2844 ^PT^	* P. savastanoi* pv. *savastanoi* ICMP4352 ^PT^	* P. syringae* DSM10604 ^T^
*** P. viridiflava* DSM6694 ^T^**	100.000 %	82.765 %	82.598 %	82.915 %	82.431 %	82.849 %	82.915 %	83.223 %	83.054 %	82.930 %	82.854 %	83.070 %	82.840 %	82.893 %
*** P. ficuserectae* ICMP7848 ^T^**	82.941 %	100.000 %	89.044 %	97.417 %	85.447 %	97.367 %	97.458 %	87.297 %	85.667 %	97.430 %	97.453 %	87.132 %	97.438 %	88.623 %
***P. caricapapayae ICMP2855* ^T^**	82.606 %	89.020 %	100.000 %	89.094 %	84.743 %	89.073 %	89.149 %	86.382 %	85.136 %	89.232 %	89.154 %	86.215 %	89.135 %	87.947 %
***P. amygdali pv. loropetali* DSM *105780* ^PT^**	82.831 %	97.385 %	89.151 %	100.000 %	85.629 %	98.510 %	98.572 %	87.483 %	86.026 %	98.200 %	98.549 %	87.255 %	98.566 %	88.725 %
***P. tremae ICMP9151* ^T^**	82.362 %	86.036 %	84.979 %	86.405 %	100.000 %	86.187 %	86.354 %	86.150 %	85.667 %	86.402 %	86.390 %	86.008 %	86.308 %	85.142 %
***P. amygdali ICMPP3918* ^T^**	82.867 %	97.433 %	89.143 %	98.668 %	85.462 %	100.000 %	98.773 %	87.393 %	85.776 %	98.312 %	98.741 %	87.129 %	98.752 %	88.723 %
***P. amygdali pv. ciccaronei ICMP5710* ^PT^**	82.911 %	97.396 %	89.185 %	98.555 %	85.717 %	98.627 %	100.000 %	87.571 %	86.324 %	98.234 %	99.459 %	87.237 %	99.585 %	88.696 %
***P. avellanae JCM11937* ^T^**	83.259 %	87.293 %	86.454 %	87.608 %	86.247 %	87.363 %	87.463 %	100.000 %	87.028 %	87.773 %	87.624 %	95.511 %	87.453 %	86.318 %
***P. cannabina ICMP2823* ^T^**	83.171 %	85.764 %	85.318 %	86.420 %	86.269 %	85.991 %	86.190 %	87.155 %	100.000 %	86.433 %	86.418 %	86.892 %	86.070 %	85.469 %
***P. amygdali pv. ulmi CFBP1407* ^PT^**	82.975 %	97.427 %	89.240 %	98.223 %	85.668 %	98.157 %	98.281 %	87.620 %	86.248 %	100.000 %	98.289 %	87.191 %	98.326 %	88.780 %
***P. savastanoi pv. fraxini CFBP5062* ^PT^**	82.835 %	97.348 %	89.160 %	98.494 %	85.672 %	98.492 %	99.423 %	87.566 %	86.148 %	98.237 %	100.000 %	87.148 %	99.663 %	88.631 %
***P. syringae pv. tomato ICMP2844* ^PT^**	83.133 %	87.009 %	86.223 %	87.210 %	86.023 %	87.087 %	87.238 %	95.485 %	86.719 %	87.250 %	87.173 %	100.000 %	87.210 %	86.299 %
***P. savastanoi pv. savastanoi ICMP4352* ^PT^**	82.883 %	97.431 %	89.206 %	98.668 %	85.516 %	98.659 %	99.608 %	87.395 %	85.858 %	98.358 %	99.774 %	87.206 %	100.000 %	88.621 %
***P. syringae DSM10604* ^T^**	82.787 %	88.582 %	87.860 %	88.804 %	84.898 %	88.608 %	88.767 %	86.098 %	85.420 %	88.874 %	88.717 %	86.080 %	88.720 %	100.000 %

PT: pathotype strain.

T: type strain.

Previously published reports [2, 9] place the pathogen closely in two different species: *P. amygdali* and *P. savastanoi,* respectively. The highest pairwise ANI of the *P. amygdali* pv. *loropetali* DSM 105780^PT^ was found with *P. amygdali* pv. *ciccaronei* ICMP 5710. However, we cannot deny the closeness of the strain in study with the pathovars of *P. savastanoi *. Previous discussions considering the high ANIs among *P. savastanoi, P. ficuserectae* and *P. amygdali* support the claim that *P. savastanoi* and *P. ficuserectae* are synonyms of *P. amygdali* [10]. Given that *P. savastanoi* is a later synonym of *P. amygdali *, and the relatedness based on ANI, we believe that the evidence is sufficient that the pathogen should continue to reside in *Pseudomonas amygdali* as pathovar *loropetali*.

## Footnotes

https://github.com/drpowell/utils/blob/master/filter-spades.py

https://github.com/FelixKrueger/TrimGalore

https://github.com/widdowquinn/pyani/blob/master/README_v_0_2_x.md
